# Editorial: Metabolic Adaptation to Cell Growth and Proliferation in Normal and Pathological Conditions

**DOI:** 10.3389/fendo.2017.00362

**Published:** 2017-12-21

**Authors:** Albert Giralt, Lluis Fajas

**Affiliations:** ^1^University of Lausanne, Lausanne, Switzerland

**Keywords:** Warburg effect, cancer metabolism, macropinocytosis, cell cycle proteins, epigenetic modifications, mitochondrial diseases, *N*-acetylaspartate, tumor microenvironment

**Editorial on the Research Topic**

**Metabolic Adaptation to Cell Growth and Proliferation in Normal and Pathological Conditions**

Proliferating cells must adapt their metabolism to fulfill the increased requirements for energy demands and biosynthetic intermediates. This adaptation is particularly relevant in cancer, where sustained rapid proliferation combined with the harsh conditions of the tumor microenvironment represent a major metabolic challenge. Noteworthy, metabolic reprogramming is now considered one of the hallmarks of cancer (1). However, the one size fits all rarely applies to the metabolic rewiring occurring in cancer cells, which ultimately depends on the combination of several factors such as the tumor’s origin, the specific genetic alterations and the surrounding microenvironment (2). In the present Research Topic, we compile a series of articles that discuss different metabolic adaptations that proliferating cells undergo to sustain growth and division, as well as the potential therapeutic window to treat certain pathologies, with a special focus on cancer.

One of the most common and well-described metabolic adaptations occurring in cancer cells is the so-called Warburg effect, which consists on high rates of glycolysis and lactate export, even in the presence of oxygen (3). Abdel-Haleem et al. show that this metabolic phenotype, far from being an exclusive feature of tumors, is a common characteristic of the proliferative state and also a usual metabolic adaptation when robust transient responses are required. Interestingly, when Otto Warburg described this phenomenon almost a century ago, he proposed that the exacerbated aerobic glycolysis observed in cancer cells was due to defective mitochondria. However, as Herst and collaborators highlight in an extensive review about the role of mitochondria in health and disease, these organelles are not only usually functional in cancer cells but also all the more essential to generate metabolic intermediates for biosynthesis, to maintain redox balance and to trigger signaling pathways that promote cell growth and proliferation.

Cell cycle progression, cellular division, and metabolism are intricate processes that regulate each other. One of the mechanisms by which proliferating cells orchestrate these phenomena in a timely manner is by the use of the cell cycle machinery to control metabolism (4). On this subject, Denechaud et al. describe how the transcription factor E2F1 couples the progression of cell cycle with the expression of genes involved in several metabolic pathways and show that dysregulation of E2F1 activity contributes to the pathophysiology of metabolic disorders such as obesity and type 2 diabetes. Another emerging link between metabolism and proliferation is the epigenetic regulation of gene expression, which is treated here in two articles. On the one hand, Rabhi and collaborators and collaborators discuss how, in response to the nutritional status, variations in the intracellular levels of certain metabolites are sensed by epigenetic cofactors that in turn promote changes in gene expression. On the other hand, Bogner-Strauss comments on the very recent literature about the novel roles of the metabolite *N*-acetylaspartate in lipogenesis and cancer progression, which include, but are not limited to, epigenetic modulation.

In the recent years, the importance of the interactions between tumor cells and their surrounding microenvironment has become clear (5). Two reviews describe different strategies developed by tumors to acquire external nutrients to sustain biomass production. Recounvreux et al. highlight the relevance of macropinocytosis as a protein source for cancer cells under nutrient-deprived conditions, whereas Blücher et al. show how lipids and other molecules delivered by adipocytes fuel tumor growth in breast cancer, unveiling a possible link with obesity.

One of the most important aspects about the study of the metabolic adaptations occurring during proliferation is the possibility of developing novel therapies to treat cancer. Fendt discusses in an opinion article the opportunities, but also the challenges, for metabolism-based anticancer strategies.

Overall, in the present Research Topic, we cover some of the different metabolic adaptations that take place during proliferation and show that they ultimately depend on both internal and external cues (cell type, history, metabolic context, etc.). Importantly, understanding the specific metabolic profile of proliferating cells may contribute to the identification of metabolic vulnerabilities in the tumors that could be exploited to increase the efficacy of the current treatments.

## Author Contributions

AG wrote the Editorial and LF edited it.

## Conflict of Interest Statement

The authors declare that the research was conducted in the absence of any commercial or financial relationships that could be construed as a potential conflict of interest.
